# Intensity-specific physical activity and mortality in 147,414 adults: a pooled accelerometer analysis

**DOI:** 10.1186/s12966-026-01980-3

**Published:** 2026-09-15

**Authors:** Michael J. Stein, Claas Lendt, Fabian Bamberg, Janett Barbaresko, Hansjörg Baurecht, Klaus Berger, Patricia Bohmann, Julian Brummer, Christoph Buck, Beate Fischer, Heinz Freisling, Daniela Georges, Karin Halina Greiser, Jana-Kristin Heise, Florian Herbolsheimer, Marie-Theres Huemer, André Karch, Thomas Keil, Lilian Krist, Wolfgang Lieb, Claudia Meinke-Franze, Rafael Mikolajczyk, Katharina Nimptsch, Nadia Obi, Cara Övermöhle, Tobias Pischon, Steffen Ringhof, Börge Schmidt, Markus Scholz, Peggy Sekula, Karen Steindorf, Paula Stürmer, Henry Völzke, Ronny Westerman, Kerstin Wirkner, Johanna Wirler, Annette Peters, Michael F. Leitzmann

**Affiliations:** 1 Department of Epidemiology and Preventive Medicine, University of Regensburg, Franz-Josef-Strauss-Allee 11, Regensburg, 93053 Germany; 2 Institute of Epidemiology, Helmholtz Zentrum München, German Research Center for Environmental Health (GmbH), Ingolstädter Landstraße 1, Neuherberg, 85764 Germany; 3 Department of Diagnostic and Interventional Radiology, Faculty of Medicine, Medical Center - University of Freiburg, University of Freiburg, Hugstetter Str. 55, 79106 Freiburg, Germany; 4 Institute for Biometrics and Epidemiology, German Diabetes Center, Leibniz Centre for Diabetes Research at Heinrich Heine University Düsseldorf, Auf’m Hennekamp 65, Düsseldorf, 40225 Germany; 5 Medical Sociology, Department of Epidemiology and Preventive Medicine, University of Regensburg, Franz-Josef-Strauss-Allee 11, Regensburg, 93053 Germany; 6 Institute of Epidemiology and Social Medicine, University of Münster, Domagkstr. 3, Münster, 48149 Germany; 7 Division of Physical Activity, Cancer Prevention and Survivorship, German Cancer Research Center (DKFZ), Im Neuenheimer Feld 581, Heidelberg, 69120 Germany; 8 Medical Faculty, Heidelberg University, Im Neuenheimer Feld 672, Heidelberg, 69120 Germany; 9 Leibniz Institute for Prevention Research and Epidemiology – BIPS, Achterstraße 30, Bremen, 28359 Germany; 10 Nutrition and Metabolism Branch, International Agency for Research on Cancer (IARC), 25 Avenue Tony Garnier, CS90627, Lyon, 69366 France; 11 Department of Child and Adolescent Psychiatry, University Medical Centre Rostock, Gehlsheimer Strasse 20, Rostock, 18147 Germany; 12 German Center for Child and Adolescent Health (DZKJ), Partner Site Greifswald/Rostock, Gehlsheimer Strasse 20, Rostock, 18147 Germany; 13 Division of Cancer Epidemiology, German Cancer Research Center, Im Neuenheimer Feld 581, Heidelberg, 69120 Germany; 14 Department of Epidemiology, Helmholtz Centre for Infection Research, Inhoffenstraße 7, Braunschweig, 38124 Germany; 15 Institute of Social Medicine, Epidemiology and Health Economics, Charité - Universitätsmedizin Berlin, Luisenstraße 57, Berlin, 10117 Germany; 16 Institute of Clinical Epidemiology and Biometry, University of Würzburg, Am Schwarzenberg 15, Würzburg, 97078 Germany; 17 State Institute of Health I, Bavarian Health and Food Safety Authority, Eggenreuther Weg 43, Erlangen, 91058 Germany; 18 Institute of Epidemiology, Kiel University, University Hospital Schleswig Holstein, Campus Kiel, Niemannsweg 11, 24105 Kiel, Germany; 19 Community Medicine, University Medicine Greifswald, Walther-Rathenau-Str. 48, Greifswald, 17475 Germany; 20 Institute of Medical Epidemiology, Biometrics, and Informatics, Medical Faculty of the Martin-Luther-University Halle-Wittenberg, Magdeburger Straße 8, Halle (Saale), 0611206112 Germany; 21 Molecular Epidemiology Research Group, Max Delbrück Center for Molecular Medicine in the Helmholtz Association (MDC), Robert-Rössle-Str. 10, Berlin, 13125 Germany; 22 Institute for Occupational and Maritime Medicine (ZfAM), University Medical Center Hamburg-Eppendorf, Martinistr. 52, Hamburg, 20246 Germany; 23 Institute for Medical Informatics, Biometry and Epidemiology, University Hospital of Essen, University of Duisburg-Essen, Zweigertstr. 37, Essen, 45130 Germany; 24 Institute for Medical Informatics, Statistics and Epidemiology, University of Leipzig, Härtelstraße 16-18, Leipzig, 04107 Germany; 25 Institute of Epidemiology and Prevention, Medical Center and Faculty of Medicine - University of Freiburg, Hugstetter Str. 49, Freiburg, 79106 Germany; 26 Federal Institute for Population Research, Friedrich-Ebert-Allee 4, Wiesbaden, 65184 Germany; 27 LIFE Research Center for Civilization Diseases, University of Leipzig, Philipp-Rosenthal-Straße 27, Leipzig, 04103 Germany; 28 Chair of Epidemiology, Institute for Medical Information Processing, Biometry and Epidemiology, Medical Faculty, Ludwig-Maximilians-University Munich, Marchioninistr. 15, Munich, 81377 Germany; 29 Partner Site Munich Heart Alliance, DZHK (German Centre for Cardiovascular Research), Munich, Germany

**Keywords:** Accelerometry, Mortality, NAKO, UK Biobank, Physical activity intensity

## Abstract

**Background:**

Insufficient physical activity is a leading global cause of premature mortality, yet how light-, moderate-, and vigorous-intensity activity differ in their associations with mortality and the implications of reallocating time across intensities remain unclear.

**Methods:**

We prospectively analysed individually pooled baseline device-measured physical activity from 147,414 participants aged 40–74 years in the German National Cohort (NAKO) and UK Biobank, the largest pooled accelerometer dataset to date. Multivariable Cox models estimated hazard ratios (HR) and 95% confidence intervals (CI) for mortality across activity intensities and under time-reallocation models holding total active time constant.

**Results:**

Over a mean follow-up of 7.9 years, 5,545 deaths occurred. Compared with no activity, 150 min per week of moderate-intensity activity was associated with lower mortality (HR: 0.70; 95% CI: 0.67, 0.72). Similar associations were observed for approximately 510 min per week of light-intensity activity or 12 min per week of vigorous-intensity activity. Additional activity beyond these levels was associated with further risk reduction. Replacing 15–60 min per week of light- or moderate-intensity activity with vigorous-intensity activity was associated with 18–30% lower mortality risk.

**Conclusions:**

Physical activity at all intensities was associated with lower mortality, with vigorous-intensity activity showing strong inverse associations even at low volumes. Shifting modest amounts of active time toward higher intensities was associated with further risk reductions. These findings provide quantitative evidence to inform the interpretation of physical activity recommendations.

**Supplementary Information:**

The online version contains supplementary material available at https://doi.org/10.1186/s12966-026-01980-3.

## Background

Insufficient physical activity affects 31% of adults worldwide and accounts for approximately 5.3 million deaths annually, making it a leading risk factor for premature mortality [1, 2]. To reduce this burden, current World Health Organization guidelines recommend 150–300 min per week of moderate-intensity activity, 75–150 min of vigorous-intensity activity, or an equivalent combination [3]. The 2:1 weighting of moderate and vigorous activity comes from metabolic equivalent of task (MET)-based classifications used in exercise physiology. These classifications were carried into self-report studies, where vigorous activity is assumed to require twice the per-minute energy expenditure of moderate activity [4].

However, this convention is not supported by accelerometer data. Device-assessed vigorous activity shows mortality reductions several-fold greater per minute than device-assessed moderate activity [5–10], far exceeding the 2:1 weighting in current guidelines [3]. Yet important uncertainties remain regarding how device-measured light-, moderate-, and vigorous-intensity activity compare quantitatively in relation to mortality across the full activity spectrum and within the active-time window. Existing studies have contrasted moderate and vigorous activity using category-based risk estimates [7, 9] Several investigations have merged these intensities into single measures, limiting direct comparison of their individual associations with mortality [11–14]. Additional work has focused exclusively on light activity [15, 16], converted activity into measures of energy expenditure rather than minutes [17], or examined time shifts between sedentary time and active behaviours [18–20]. These approaches do not isolate shifts within active time, where interpretation of intensity weightings is clearest.

To address these questions, we aimed to provide directly interpretable, minute-based comparisons of activity intensities in relation to mortality and to estimate the impact of shifting time toward higher intensities within the active-time window. We analysed device-measured physical activity from two large prospective studies: the German National Cohort (NAKO Gesundheitsstudie; NAKO) with hip-worn accelerometers and UK Biobank (UKB) with wrist-worn devices. Using two complementary populations and assessment methods enabled us to evaluate the consistency of results across cohorts, enhancing the robustness of the observed associations. To our knowledge, this is the largest accelerometer-based analysis to examine mortality associations across light, moderate, and vigorous activity.

## Methods

### Study populations

NAKO is a prospective cohort of adults aged 19–74 years recruited across 18 study centres in Germany between 2014 and 2019, with a baseline response proportion of 15.6% [21]. Among 205,415 participants, 72,553 wore a hip-mounted accelerometer.

UKB is a prospective cohort of adults aged 40–69 years recruited across 22 study centres in the UK between 2006 and 2010, with a baseline response proportion of 5.5% [22]. Among 502,383 participants, 103,578 wore a dominant-wrist accelerometer.

All participants provided written informed consent; detailed descriptions are available elsewhere [23, 24]. Ethics approvals were granted by the relevant local committees. We excluded individuals with inadequate accelerometer data (insufficient wear time, invalid recordings; NAKO, *n* = 7,431; UKB, *n* = 6,984), those lacking follow-up information in NAKO (*n* = 195), and those lost in UKB (*n* = 4), yielding 147,414 participants (36% from NAKO) for analysis (Supplementary Fig. 1).

### Physical activity assessment

In NAKO, physical activity was assessed at baseline using ActiGraph accelerometers (GT3X+, wGT3X+, wGT3X-BT; ActiGraph, Pensacola, FL, USA) for seven consecutive days. Raw 100-Hz data were processed using GGIR (v3.2-9) [25, 26]. In UKB, physical activity was measured between 2013 and 2015 using Axivity AX3 accelerometers (Axivity Ltd, Newcastle, UK) for seven consecutive days. Raw 100-Hz data were processed using methods also applied in NAKO [27]. We used identical wear time criteria in both cohorts, requiring at least 72 h of valid recordings across the 24-hour cycle.

Overall activity volume was quantified as average weekly acceleration (Euclidean Norm Minus One, ENMO; milli-*g*). Physical activity intensity was defined as weekly minutes spent in light, moderate, and vigorous activity, using cut-points of 10, 69, and 259 milli-*g* for hip-worn accelerometers used in NAKO and 30, 100, and 420 milli-*g* for wrist-worn accelerometers used in UKB, respectively [28, 29]. Given inconsistencies in accelerometer cut-points [29–33] and implausibly high estimates produced by some calibration approaches [34–36], we used a conservative light activity cut-point [28]. These thresholds correspond approximately to increasing levels of ambulatory movement intensity but should not be interpreted as exact behavioral equivalents because calibration varies across populations and protocols. Activity variables were winsorized at the 99.9th percentile.

### Cohort follow-up and mortality ascertainment

In NAKO, vital status was ascertained through 30 April 2024 using both active and passive ascertainment procedures, including participant contact, registry linkage, and death certificate verification [37]. Follow-up extended from the accelerometry assessment (April 2014 to August 2019) until death or censoring at the last known vital status, with additional deaths identified through supplementary notifications recorded up to December 2025.

In UKB, vital status was obtained through linkage with routine health-care data and national death registries [38]. Follow-up began at the accelerometry assessment (June 2013 to December 2015) and continued until death, loss-to-follow-up, or end of follow-up (30 November 2022) [39].

The endpoint was all-cause mortality.

### Covariates

Potential confounders were selected using directed acyclic graphs [40] (Supplementary Fig. 2) and harmonized across cohorts. Covariate details are provided in Table 1. In brief, covariates included sex, age group, study region, educational level, socioeconomic status, employment status, alcohol consumption, smoking status, prevalent chronic disease (cardiovascular disease, diabetes, or cancer), body mass index, handgrip strength, sedentary behaviour, and sleep duration.

**Table 1:** Age-standardised baseline characteristics of participants of the German National Cohort (NAKO, *N* = 52,221) and UK Biobank (*N* = 95,193) by tertiles of total physical activity

Characteristic	NAKO			UK Biobank		
	1st tertile	2nd tertile	3rd tertile	1st tertile	2nd tertile	3rd tertile
N	17,407	17,407	17,407	31,488	31,835	31,870
Average acceleration (milli-*g*)	8.8 (2.4)	11.6 (2.7)	14.4 (3.5)	20.6 (4.4)	27.5 (4.5)	35.3 (6.9)
Light physical activity (min/week)	1250.9 (235.8)	1714.5 (188.7)	2348.7 (449.9)	1443.2 (248.3)	1816.0 (209.2)	2164.2 (300.5)
Moderate physical activity (min/week)	274.8 (125.6)	383.1 (138.9)	497.3 (284.0)	471.0 (176.8)	712.1 (188.9)	1012.0 (289.5)
Vigorous physical activity (min/week)	8.5 (27.4)	14.5 (38.5)	16.7 (42.2)	14.4 (25.2)	25.0 (33.9)	38.3 (44.3)
Wear time (h/day)	23.8 (0.4)	23.8 (0.4)	23.7 (0.4)	22.8 (2.3)	22.9 (2.2)	22.8 (2.3)
Sex						
Men	48.8	45.2	49.7	54.0	43.0	34.5
Women	51.2	54.8	50.3	46.0	57.0	65.5
Age (years)	55.4 (8.1)	54.1 (7.8)	53.2 (7.4)	64.0 (5.9)	63.0 (6.1)	62.2 (6.3)
Educational level^1^						
Highest	57.7	59.1	52.1	42.7	43.9	41.6
Intermediate	39.4	38.7	45.0	24.2	23.7	24.0
Lowest	2.8	2.2	2.8	22.7	24.0	26.1
Other	0.1	0.1	0.1	10.4	8.4	8.3
Missing	0.1	0.0	0.1	1.1	0.9	0.9
Employment status						
Employed	70.7	79.6	83.7	58.1	63.3	67.1
Unemployed	4.1	2.2	1.7	1.4	1.1	1.1
Not in labour force	25.2	18.2	14.6	40.5	35.6	31.8
Missing	0.7	0.6	0.6	0.7	0.7	0.8
Socio-economic status^2^	51.7 (15.6)	51.1 (15.6)	47.0 (16.2)	-1.6 (3.0)	-1.8 (2.8)	-1.9 (2.8)
Missing	4.2	3.6	2.9	0.1	0.1	0.1
Body mass index (kg/m^2^)	27.8 (5.6)	26.4 (4.7)	26.2 (4.4)	28.0 (5.1)	26.6 (4.4)	25.6 (4.1)
Missing	2.4	1.9	1.8	0.4	0.2	0.1
Grip strength (kg)	38.4 (12.0)	38.6 (11.7)	39.6 (12.0)	34.3 (11.8)	32.9 (11.5)	31.5 (10.8)
Missing	1.9	1.4	1.3	0.2	0.2	0.2
Sedentary behaviour (h/day)	7.8 (4.1)	6.7 (3.8)	5.6 (3.6)	4.9 (2.6)	4.2 (2.4)	3.8 (2.4)
Missing	8.6	7.7	8.0	0.1	0.1	0.1
Sleep duration (h/day)	7.9 (1.3)	7.8 (1.1)	7.6 (1.2)	7.2 (1.1)	7.2 (1.0)	7.1 (1.0)
Missing	11.8	10.7	13.8	0.4	0.2	0.3
Alcohol consumption (g/day)	10.5 (17.0)	10.8 (17.0)	11.3 (17.2)	17.1 (20.3)	16.4 (18.1)	15.5 (17.5)
Missing	3.3	2.7	3.2	14	12.2	12.2
Smoking status						
Never	43.2	47.7	46.5	53.8	57.1	57.8
Former	36.2	35.5	36.0	38.0	37.0	36.3
Current	20.5	16.8	17.5	8.2	5.9	5.8
Missing	2.7	2.2	2.7	0.3	0.3	0.3
Cardiovascular diseases^3^						
No	81.7	85.9	87.6	93.2	96.0	97.2
Yes	18.3	14.1	12.4	6.8	4.0	2.8
Missing	0.0	0.0	0.0	0.0	0.0	0.0
Diabetes^3^						
No	91.4	94.8	95.5	98.0	99.1	99.5
Yes	8.6	5.2	4.5	2.0	0.9	0.5
Missing	0.2	0.2	0.2	0.0	0.0	0.0
Cancer^3^						
No	91.2	92.5	93.9	92.5	93.1	93.3
Yes	8.8	7.5	6.1	7.5	6.9	6.7
Missing	0.4	0.3	0.3	0.0	0.0	0.0

Figures are age-standardised mean (standard deviation) or percentages. Age in years was not standardized. Age-standardization was performed by applying survey weights so that the age-group distribution within each physical activity tertile matched the overall age-group distribution of the study population

Observed interquartile ranges of physical activity in NAKO: Average acceleration: [9.0; 13.4] milli-g. Light physical activity: [1397; 2081] minutes/week. Moderate physical activity: [256; 477] minutes/week. Vigorous physical activity: [0.3; 5.9] minutes/week

Observed ranges of physical activity in UK Biobank: Average acceleration: [22.4; 32.5] milli-g. Light physical activity: [1542; 2046] minutes/week. Moderate physical activity: [524; 927] minutes/week. Vigorous physical activity: [10; 30] minutes/week

^1^Educational levels were defined as first stage of tertiary education/second stage of tertiary education (highest); upper secondary level of education/post-secondary, non-tertiary education (intermediate); primary level of education/lower secondary level of education (lowest); still in vocational training (other) in NAKO; and college or university (highest); A/AS, NVQ/HND/HNC or equivalent, other qualifications (intermediate); O/GCSEs, CSEs or equivalent (lowest); none of those (other) in UK Biobank

^2^Socio-economic status was defined by the International Socio-economic Index of Occupational Status (NAKO; higher values indicate lower deprivation) or Townsend Index of Deprivation (UK Biobank; higher values indicate higher deprivation)

^3^Prevalent chronic diseases were obtained from self-reported physician diagnoses (NAKO) and hospital inpatient record (UK Biobank)

### Statistical analysis

Participant characteristics were summarized using means (standard deviations) or counts (%). Correlations between activity measures were examined using Spearman correlation. Missing covariates were handled via multiple imputation by chained equations (ten datasets, 5 iterations) using predictive mean matching for continuous variables and appropriate regression models for categorical variables [41].

We investigated the relations of physical activity volume and intensity to mortality using Cox regression with age as the time metric, estimating hazard ratios (HR) and 95% confidence intervals (CI). Proportional hazards assumptions were verified using Schoenfeld residuals and visual inspection; with no violations detected. We performed separate analyses for each cohort and additionally fitted individual-level pooled models. For pooled analyses, cohort-specific variables were z-standardised, and cohort was included as a stratification term.

First, in separate, cohort-specific models, we modelled weekly minutes of light, moderate, and vigorous activity as continuous variables. Non-linearity was addressed using restricted cubic splines with knots at the 0.10, 0.50, 0.90 quantiles [42]. Intensity equivalence was determined by estimating the durations of light and vigorous activity yielding the same HR as 150 min of moderate activity. Equivalence estimates were restricted to observed activity ranges and reflect model-based rather than physiological equivalence.

Second, based on the estimated parameters, we examined potential reallocations of active time between intensities while holding total active time constant, thereby reflecting reallocations within active time. Intensities were scaled per 30 min, and models were adjusted for total activity to estimate effects of reallocating 15, 30, 45, and 60 min per week between intensities. To be precise, following the isotemporal substitution approach described by Galmes-Panades et al. [43], time spent in each intensity was divided by 30 and the model for re-allocating light-intensity activity was mutually adjusted for moderate and vigorous activity, along with total physical activity (sum of time spent in each intensity). The HRs represent the effect of theoretically substituting 0.5, 1, 1.5, and 2 units (15–60 min) of light-intensity activity with that from another intensity, rotating the reference intensity to obtain results for each.

Third, we classified moderate activity into four categories defined by minutes per week: <75, 75–149, 150–300, and ≥ 300. Because exposure distributions differed across cohorts, vigorous activity was classified into harmonized distribution-based categories defined by zero minutes per week, below-median activity, and at-or-above median activity, all in minutes per week; the median was defined among participants reporting any vigorous activity.

Sensitivity analyses excluded early follow-up (1–5 years), removed potential mediators from the adjustment set (body mass index, grip strength, chronic diseases), restricted analyses to participants without prevalent chronic disease and never smokers, additionally adjusted for season of accelerometer wear, and mutually adjusted activity intensities.

Two-sided P values < 0.05 were considered statistically significant. Analyses were conducted in R version 4.2.3 [44]. Cox models were constructed with the *rms* package^45^, multiple imputation was performed with *mice*^41^.

## Results

### Baseline characteristics

Across activity gradients, among 147,414 participants (55% women; median age 60 years), higher overall activity was generally associated with more favourable health characteristics, including lower body mass index, less sedentary time, less current smoking, fewer chronic diseases, and a greater likelihood of employment (Table 1, Supplementary Table 1).

Higher total activity in NAKO was associated with slightly lower socioeconomic status, higher alcohol consumption, and higher grip strength, whereas in UKB, higher activity was characterized by lower socioeconomic deprivation, lower alcohol consumption, a greater proportion of women, and lower grip strength.

NAKO participants were more likely to have the highest educational attainment, reported lower alcohol consumption, and had more sedentary time, whereas UKB participants were more often female, less frequently employed, and spent more time in higher-intensity activity.

### Correlations between physical activity metrics

Overall activity volume showed moderate to strong correlations with each intensity category (Spearman *r* = 0.54–0.72), reflecting that time spent at any intensity contributed to total volume. Light activity correlated only weakly with moderate (*r* = 0.34) and vigorous activity (*r* = 0.13), whereas moderate activity and vigorous activity were more closely correlated (*r* = 0.60) (Supplementary Fig. 3).

### Relations of physical activity volume and intensity to mortality

**Fig. 1:**
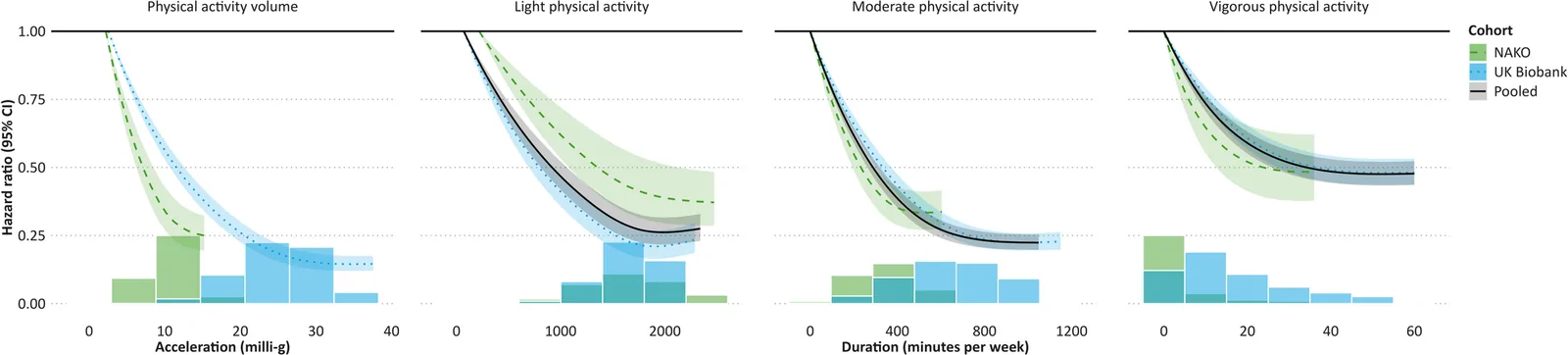
Physical activity volume and intensity in relation to mortality. All P for non-linearity < 0.05. Restricted cubic splines used knots at the 10th, 50th and 90th percentiles. Models were adjusted for categorical variables including sex, age group (5-year increments), study region (NAKO: south-east, south-west, north-east, north-west, west, central Germany, and Berlin; UK Biobank: England, Scotland, Wales), educational level (low, intermediate, high, other), employment status (employed; unemployed; outside the labour force), smoking status (never, former, current), prevalent cardiovascular disease, diabetes, or cancer (NAKO: self-reported; UK Biobank: hospital inpatient data); and continuous variables including socio-economic status (NAKO: International Socio-economic Index of Occupational Status, UK Biobank: Townsend Index of Deprivation), alcohol consumption (grams per day), measured body mass index (kg/m^2^), maximum handgrip strength (kg), sedentary behaviour (hours/day), and sleep duration (hours/day). Physical activity intensities were not mutually adjusted. The panel for physical activity volume does not show a pooled estimate because the x-axis is expressed in milli-*g* units. Pooling required standardising exposures to z-scores, which are not directly interpretable. For visualization purposes, the x-axis was truncated at the 90th percentile of the distribution of each physical activity exposure. Shaded areas represent 95% CIs, solid lines represent HRs, and rug plots show the distribution of participants

In pooled analyses, mean follow-up was 7.9 years (1,170,697 person-years), during which 5,545 deaths occurred (29% in NAKO). In multivariable-adjusted analyses, higher total activity volume was associated with lower mortality, with HRs decreasing steeply at low activity levels and plateauing thereafter (Fig. 1). All activity intensities were inversely associated with mortality, but dose–response shapes differed: vigorous activity showed the greatest risk reduction per minute, followed by moderate and light activity. Patterns were similar across cohorts, although associations for light activity were slightly weaker and those for vigorous activity slightly stronger in NAKO than UKB.

When comparing intensities directly, approximately 510 min of light activity or 12 min of vigorous activity per week yielded mortality reductions comparable to 150 min of moderate activity (multivariable-adjusted HR for 150 min/week of moderate activity: 0.70; 95% CI: 0.67, 0.72), compared to no activity.

### Reallocating time between intensities in relation to mortality

**Fig. 2:**
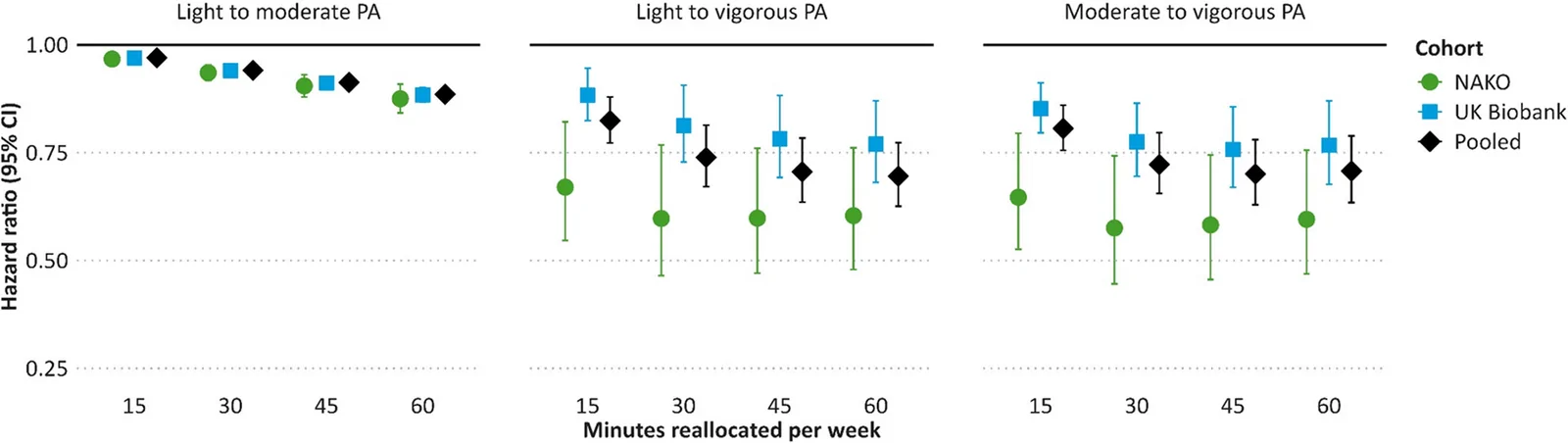
Hypothetical reallocation of time between activity intensities in relation to mortality (total volume constant). Models were adjusted for categorical variables including sex, age group (5-year increments), study region (NAKO: south-east, south-west, north-east, north-west, west, central Germany, and Berlin; UK Biobank: England, Scotland, Wales), educational level (low, intermediate, high, other), employment status (employed; unemployed; outside the labour force), smoking status (never, former, current), prevalent cardiovascular disease, diabetes, or cancer (NAKO: self-reported; UK Biobank: hospital inpatient data); and continuous variables including socio-economic status (NAKO: International Socio-economic Index of Occupational Status, UK Biobank: Townsend Index of Deprivation), alcohol consumption (grams per day), body mass index (kg/m^2^), maximum handgrip strength (kg), sedentary behaviour (hours/day), and sleep duration (hours/day). Physical activity intensities were not mutually adjusted. Error bars represent 95% CIs, solid dots represent HRs. PA: physical activity

In multivariable-adjusted analyses, replacement of lower with higher activity intensity was associated with progressively lower mortality (Fig. 2). Reallocating 15, 30, 45, or 60 min per week from light to moderate activity corresponded to approximately 3%, 6%, 9%, and 11% lower mortality, respectively. Shifting time from either light or moderate activity to vigorous activity yielded larger reductions, ranging from about 18% to 30%, with stronger relations in NAKO than in UKB.

### Categories of moderate and vigorous activity in relation to mortality

**Fig. 3:**
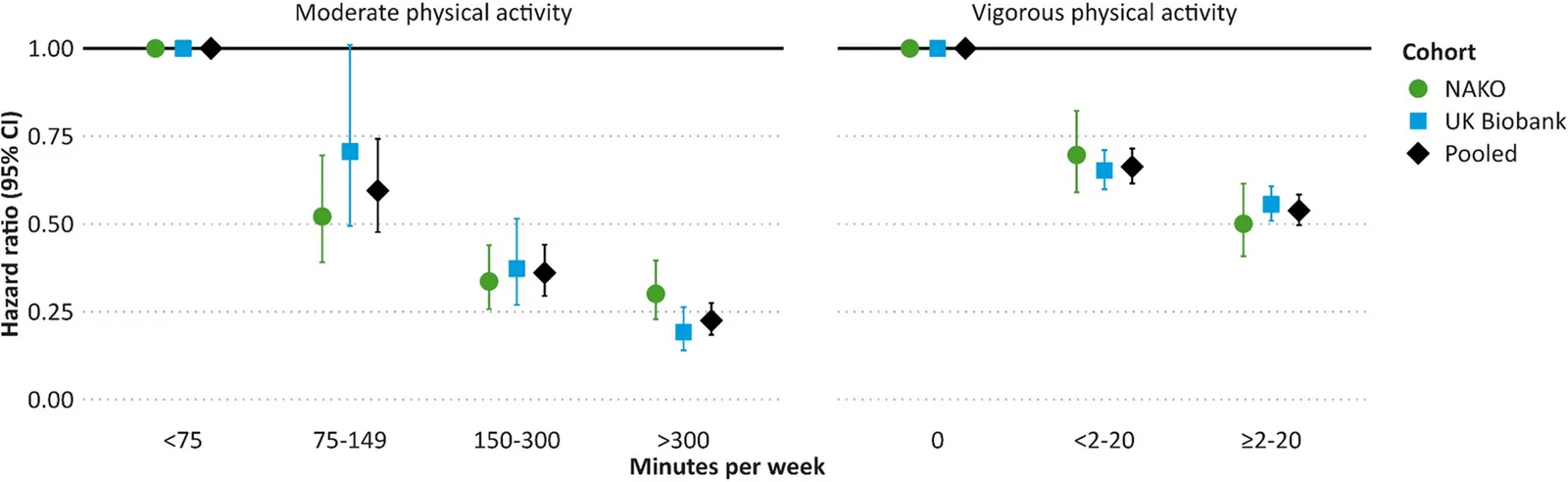
Categories of moderate and vigorous activity in relation to mortality. Models were adjusted for categorical variables including sex, age group (5-year increments), study region (NAKO: south-east, south-west, north-east, north-west, west, central Germany, and Berlin; UK Biobank: England, Scotland, Wales), educational level (low, intermediate, high, other), employment status (employed; unemployed; outside the labour force), smoking status (never, former, current), prevalent cardiovascular disease, diabetes, or cancer (NAKO: self-reported; UK Biobank: hospital inpatient data); and continuous variables including socio-economic status (NAKO: International Socio-economic Index of Occupational Status, UK Biobank: Townsend Index of Deprivation), alcohol consumption (grams per day), body mass index (kg/m^2^), maximum handgrip strength (kg), sedentary behaviour (hours/day), and sleep duration (hours/day). Physical activity intensities were not mutually adjusted. Error bars represent 95% CIs, solid dots represent HRs. The median of > 0 min of vigorous physical activity was 1.6 and 20.2 min in NAKO and UK Biobank, respectively

Participants achieving 150–300 min per week of moderate activity had considerably lower mortality, with a 64% relative risk reduction compared to those performing less than 75 min per week. In UKB, but not in NAKO, more than 300 min per week of moderate activity was associated with further risk reduction (Fig. 3).

Even 2–20 min of vigorous activity per week (i.e., the lower vigorous activity category defined by cohort-specific distributions among those reporting any vigorous activity) was associated with substantially lower mortality risk (multivariable-adjusted HR: 0.66; 95% CI: 0.62, 0.71) compared with no vigorous activity. Higher vigorous activity was associated with additional risk reduction (multivariable-adjusted HR: 0.54; 95% CI: 0.50, 0.58) (Fig. 3).

### Sensitivity analyses

Excluding the first 1–5 years of follow-up, removing body mass index, grip strength, and chronic disease from the adjustment set, restricting analyses to participants without baseline chronic diseases and never smokers, and adjusting for season of accelerometer wear yielded similar results (Supplementary Figs. 4–8).

Mutual adjustment for activity intensities produced similar patterns. For light activity, the inverse J-shape became slightly flatter, and the association with vigorous activity was modestly attenuated (Supplementary Fig. 9).

## Discussion

In this accelerometer-based analysis of 147,414 adults from two European cohorts, higher-intensity physical activity was consistently associated with larger relative reductions in mortality. Light, moderate, and vigorous activity were each related to lower mortality, but the magnitudes differed substantially. When intensities were compared directly, approximately 510 min per week of light activity or 12 min per week of vigorous activity corresponded to mortality benefits similar to 150 min per week of moderate activity (HR = 0.70). When we examined how individuals might shift the time they already spend being active, replacing 15–60 min per week of light activity with moderate activity was associated with 3–12% lower mortality. Shifting the same amount from either light or moderate toward vigorous activity was associated with reductions of 18–30%. These patterns were broadly consistent in both cohorts.

Across analyses, reallocating time from light or moderate to vigorous activity was associated with the largest mortality reductions, indicating disproportionate associations for vigorous activity. Importantly, reallocations were examined within active time rather than between sedentary and active behaviours, isolating how the distribution of already-active minutes across intensities relates to mortality while holding total active time constant. These findings suggest that meaningful risk reductions may arise from small shifts toward higher-intensity activity, even without increasing total active time.

Complementary findings emerged from continuous dose-response analyses. Mortality declined sharply as individuals moved out of the lowest activity range, regardless of intensity, indicating that any activity is preferable to almost none. However, the decline was steeper for moderate and especially vigorous activity, suggesting stronger inverse associations at higher intensities. Categorical analyses reinforced these patterns: moderate activity showed a graded mortality relation, whereas vigorous activity behaved more like a threshold, with the greatest contrast between no and any vigorous engagement. We note that the category boundaries for vigorous activity differed between cohorts; thus, these findings should not be interpreted as indicating a minimum effective dose of vigorous-intensity physical activity, i.e., that only 2 min per week would be sufficient for a substantially lower mortality risk. Rather, the observed risk reduction was associated with the entire 2–20 min per week category, and it is likely that individuals in the upper end of this range contributed most strongly to the observed association. Taken together, these findings consistently indicate that light activity relates to lower mortality, moderate activity shows a stronger association, and even small amounts of vigorous activity are associated with additional risk reduction.

We leveraged data from two large-scale European cohorts with broadly similar characteristics, although differences existed in accelerometer devices and wear locations. Wrist-worn accelerometry may classify upper-limb movement unrelated to whole-body metabolic intensity as higher-intensity activity, potentially contributing to higher estimated volumes of moderate and vigorous activity and attenuated associations with mortality in UKB. By comparison, hip-derived activity, driven primarily by centre-of-mass locomotion, may more closely reflect ambulatory movement intensity [36], partly explaining the stronger associations observed in NAKO. Replication across cohorts is particularly important in accelerometer epidemiology because estimated activity intensities depend strongly on device type, wear location, calibration strategy, and processing decisions [29–36]. The broad consistency of our findings across hip- and wrist-derived accelerometry therefore strengthens confidence that the observed gradients are not solely artefacts of a specific device configuration or analytical pipeline. In addition, the choice of acceleration metric may contribute to measurement error. For example, ENMO, which was used in both cohorts, has been reported to exhibit body size-dependent measurement error because negative accelerations are truncated, potentially attenuating associations with health outcomes [46, 47]. Future studies using alternative processing approaches are warranted to investigate this potential source of error.

Our analyses extend earlier accelerometer-based research showing that vigorous movement is more strongly associated with mortality than moderate activity [5–10]. Previous studies reported that 15–20 min per week of vigorous physical activity was associated with 18–24% lower mortality [5] and that considerably smaller amounts of vigorous activity than moderate or light activity attenuated the sedentary time–mortality association [6]. In the study most similar to our approach, 45 min per week of vigorous, 155 min per week of moderate, and 1,380 min per week of light physical activity were estimated to achieve a 50% mortality reduction, although analyses were restricted to individuals with cardiovascular disease [7]. Similarly, equivalence analyses across physical activity intensities suggested that 4.2 min per day of moderate physical activity and 58.1 min per day of light physical activity were equivalent to 1 min per day of vigorous physical activity for a 10% risk reduction [9]. Other studies likewise reported stronger mortality associations for higher-intensity activity, including incidental vigorous activity performed during daily living [10] and moderate-to-vigorous activity among individuals with cardiometabolic disease [8].

Our findings also complement the growing literature on how physical activity is distributed across the intensity spectrum in relation to mortality. Studies using the intensity gradient [48, 49] and related accelerometer metrics [50–52] have shown that individuals who accumulate their most demanding minutes at higher intensities tend to have lower mortality. Extending this work, our analysis quantified how reallocating activity time across intensities relates to mortality while holding total active time constant. Together, these findings provide complementary perspectives on how activity intensity and the distribution of active time relate to mortality.

Several biological pathways may explain the patterns observed. Vigorous activity induces larger, more rapid increases in heart rate and ventilation than lower-intensity activity [53] and elicits stronger adaptations in cardiorespiratory fitness, endothelial function, and insulin sensitivity [54–56]. These physiological systems strongly predict mortality [57–59], and their improvement may help explain the pronounced associations observed with vigorous activity. Although accelerometers cannot capture the underlying mechanisms directly, the consistent gradient in mortality associations across intensities aligns with this physiological framework. Future studies investigating cause-specific mortality are needed to better understand the pathways underlying the observed associations and to inform more targeted public health guidance.

Our findings suggest that physical activity guidance may benefit from considering not only total activity volume but also how active time is distributed across intensities. However, these estimates reflect statistical associations derived from device-measured activity patterns and should not be interpreted as physiological equivalence between activity intensities or as a direct substitution for current physical activity recommendations. Nonetheless, encouraging brief periods of more demanding movement within daily routines, e.g., faster walking, may represent practical ways to build on existing activity patterns, particularly among individuals already achieving moderate activity levels. At the same time, the steep reductions observed when moving out of the least-active range reinforce that any increase in activity remains beneficial, regardless of intensity. Together with accumulating evidence, our study supports translating activity intensity into public health and clinical guidance.

### Strengths and limitations

This is, to our knowledge, the largest accelerometer-based analysis to compare mortality associations across light, moderate, and vigorous intensities using multiple complementary approaches. We harmonised methods across two large cohorts using different devices and wear locations, and a broad suite of sensitivity analyses supported the robustness of the findings. Limitations include the observational design, which precludes causal inference; potential residual confounding, including genetic predisposition, socioeconomic factors, and other health behaviours that may influence both physical activity behaviour and mortality risk; and the inability of accelerometers to capture contextual information about physical activity, such as distinguishing between resistance and aerobic exercise. Although we adjusted for a broad range of demographic, lifestyle, and health-related factors and findings were robust across sensitivity analyses, unmeasured confounding cannot be excluded. Reverse causation also remains possible despite exclusion of early follow-up periods, as preclinical disease or declining functional capacity may reduce participation in vigorous activity prior to death. Estimated activity intensities were based on established cut-points, but limited consensus exists regarding the optimal thresholds. Different cut-points classify activity differently and may therefore influence the estimated exposure–mortality associations. Although all-cause mortality represents an important endpoint for public health guidance, it may obscure differences across specific causes of death and does not allow inference about the underlying mechanisms. Lastly, the predominantly white European volunteer sample limits generalizability.

## Conclusion

In this study of nearly 150,000 adults, 150 min per week of moderate activity was associated with 30% lower mortality, comparable to 512 min of light or 12 min per week of vigorous activity. Hypothetically replacing 60 min per week of light activity with moderate or vigorous activity was associated with 12% and 30% lower mortality, respectively, indicating that modest shifts toward higher-intensity movement provide additional benefit. By quantifying time-based equivalences and examining reallocations within the active-time window, our study provides an integrated perspective on how activity intensities relate to mortality.

## Supplementary Information

Supplementary Material 1: STROBE checklist.

Supplementary Material 2: Supplementary Analyses.

## Data Availability

Registered researchers from all European Union (EU) countries can apply to use the NAKO dataset by registering and applying at http://transfer.nako.de/.UK Biobank is an open access resource. Bona fide researchers can apply to use the UK Biobank dataset by registering and applying at http://ukbiobank.ac.uk/register-apply/.

## Abbreviations

CI: Confidence interval
ENMO: Euclidean Norm Minus One
HR: Hazard ratio
MET: Metabolic equivalent of task
NAKO: German National Cohort (NAKO Gesundheitsstudie)
UKB: UK Biobank
