# Supplementary material for: Intensity-specific physical activity and mortality in 147,414 adults: a pooled accelerometer analysis
Source: Int J Behav Nutr Phys Act. 2026 Sep 15;23:85. doi: 10.1186/s12966-026-01980-3 (PMC13576285; doi:10.1186/s12966-026-01980-3)
Supplement: Supplementary file 1 [file 12966_2026_1980_MOESM2_ESM.docx]

Intensity-specific physical activity and mortality in 147,414 adults: a pooled accelerometer analysis

Supplementary material

[Supplementary Table 1.	Crude baseline characteristics of participants of the German National Cohort and UK Biobank by tertiles of total physical activity](#_Toc236736223)

[Supplementary Figure 1.	Flowchart of participant inclusion](#_Toc236736224)

[Supplementary Figure 2.	Directed acyclic graph for confounder selection](#_Toc236736225)

[Supplementary Figure 3.	Correlation matrix for all physical activity exposures](#_Toc236736226)

[Supplementary Figure 4.	Exclusion of 1 to 5 years of follow-up](#_Toc236736227)

[Supplementary Figure 5.	Main results without adjustment for body mass index, grip strength, cardiovascular disease, diabetes, and cancer](#_Toc236736228)

[Supplementary Figure 6.	Main results after exclusion of prevalent cardiovascular disease, diabetes, and cancer](#_Toc236736229)

[Supplementary Figure 7.	Main results after restriction to participants who never smoked](#_Toc236736230)

[Supplementary Figure 8.	Main results with additional adjustment for season of accelerometer wear](#_Toc236736231)

[Supplementary Figure 9.	Main results under mutual adjustment for light, moderate, and vigorous physical activity](#_Toc236736232)

**Corresponding author:** Michael J. Stein, Tel.: +49 89 318748819, Mail: michael.stein@helmholtz-munich.de, Department of Epidemiology and Preventive Medicine, University of Regensburg, Regensburg, Germany

## Supplementary Table 1. Crude baseline characteristics of participants of the German National Cohort and UK Biobank by tertiles of total physical activity

|  | **NAKO** | | | **UK Biobank** | | |
| --- | --- | --- | --- | --- | --- | --- |
| **Characteristic** | **T1**  N = 17,407 | **T2**  N = 17,407 | **T3**  N = 17,407 | **T1**  N = 31,488 | **T2**  N = 31,835 | **T3**  N = 31,870 |
| **Average acceleration (milli-*g*)** | 8.7 (2.4) | 11.5 (2.6) | 14.3 (3.4) | 20.8 (4.5) | 27.7 (4.6) | 35.6 (7.1) |
| **Light physical activity (min/week)** | 1,251.6 (234.1) | 1,717.0 (186.1) | 2,351.2 (444.1) | 1,438.7 (238.0) | 1,808.5 (200.8) | 2,157.2 (288.2) |
| **Moderate physical activity (min/week)** | 271.0 (124.2) | 380.7 (138.1) | 494.7 (282.9) | 477.2 (173.0) | 718.5 (181.7) | 1,023.2 (284.1) |
| **Vigorous physical activity (min/week)** | 8.1 (25.9) | 14.1 (37.0) | 16.4 (40.7) | 15.3 (27.0) | 26.5 (35.7) | 40.6 (46.4) |
| **Wear time (h/day)** | 23.8 (0.4) | 23.8 (0.4) | 23.7 (0.4) | 22.8 (2.3) | 22.9 (2.1) | 22.8 (2.3) |
| **Sex** |  |  |  |  |  |  |
| Men | 8,574 (49.3%) | 7,927 (45.5%) | 8,631 (49.6%) | 16,886 (53.6%) | 13,557 (42.6%) | 11,017 (34.6%) |
| Women | 8,833 (50.7%) | 9,480 (54.5%) | 8,776 (50.4%) | 14,602 (46.4%) | 18,278 (57.4%) | 20,853 (65.4%) |
| **Age (years)** | 56.2 (8.7) | 54.7 (8.5) | 53.6 (8.2) | 62.9 (7.6) | 61.6 (7.7) | 60.5 (7.7) |
| **Educational level** |  |  |  |  |  |  |
| Highest | 10,020 (57.6%) | 10,269 (59.0%) | 9,064 (52.1%) | 13,574 (43.6%) | 14,139 (44.8%) | 13,304 (42.1%) |
| Intermediate | 6,872 (39.5%) | 6,721 (38.6%) | 7,820 (45.0%) | 7,488 (24.0%) | 7,401 (23.5%) | 7,446 (23.6%) |
| Lowest | 494 (2.8%) | 394 (2.3%) | 495 (2.8%) | 7,085 (22.7%) | 7,630 (24.2%) | 8,468 (26.8%) |
| Other | 9 (0.1%) | 14 (0.1%) | 14 (0.1%) | 3,003 (9.6%) | 2,381 (7.5%) | 2,359 (7.5%) |
| Missing | 12 | 9 | 14 | 338 | 284 | 293 |
| **Employment status** |  |  |  |  |  |  |
| Employed | 11,669 (67.5%) | 13,338 (77.1%) | 14,158 (81.8%) | 18,972 (60.7%) | 21,157 (66.9%) | 22,311 (70.5%) |
| Unemployed | 675 (3.9%) | 366 (2.1%) | 296 (1.7%) | 463 (1.5%) | 345 (1.1%) | 369 (1.2%) |
| Not in labour force | 4,944 (28.6%) | 3,595 (20.8%) | 2,856 (16.5%) | 11,846 (37.9%) | 10,128 (32.0%) | 8,952 (28.3%) |
| Missing | 119 | 108 | 97 | 207 | 205 | 238 |
| **Socio-economic status** | 51.6 (15.0) | 51.2 (15.1) | 47.1 (15.7) | -1.6 (2.9) | -1.8 (2.8) | -1.8 (2.8) |
| Missing | 734 | 624 | 519 | 46 | 38 | 24 |
| **Body mass index (kg/m^2^)** | 27.9 (5.4) | 26.5 (4.5) | 26.2 (4.3) | 28.0 (5.0) | 26.6 (4.3) | 25.6 (4.0) |
| Missing | 431 | 334 | 320 | 113 | 56 | 44 |
| **Grip strength (kg)** | 38.2 (11.5) | 38.5 (11.4) | 39.4 (11.7) | 34.5 (11.4) | 33.2 (11.0) | 32.0 (10.5) |
| Missing | 341 | 254 | 231 | 69 | 66 | 47 |
| **Sedentary behaviour (h/day)** | 7.7 (3.8) | 6.6 (3.6) | 5.6 (3.4) | 4.9 (2.6) | 4.2 (2.3) | 3.8 (2.4) |
| Missing | 1,522 | 1,360 | 1,403 | 26 | 19 | 27 |
| **Sleep duration (h/day)** | 7.9 (1.2) | 7.8 (1.1) | 7.6 (1.1) | 7.2 (1.1) | 7.2 (1.0) | 7.1 (0.9) |
| Missing | 2,168 | 1,929 | 2,464 | 118 | 75 | 90 |
| **Alcohol consumption (g/day)** | 10.6 (16.8) | 10.9 (16.8) | 11.3 (16.8) | 16.9 (17.9) | 16.2 (16.1) | 15.5 (15.9) |
| Missing | 604 | 497 | 580 | 4,463 | 3,965 | 3,953 |
| **Smoking status** |  |  |  |  |  |  |
| Never | 7,297 (43.2%) | 8,101 (47.6%) | 7,877 (46.5%) | 17,213 (54.8%) | 18,414 (58.0%) | 18,686 (58.8%) |
| Former | 6,224 (36.8%) | 6,103 (35.9%) | 6,138 (36.3%) | 11,549 (36.8%) | 11,348 (35.7%) | 11,118 (35.0%) |
| Current | 3,385 (20.0%) | 2,802 (16.5%) | 2,909 (17.2%) | 2,639 (8.4%) | 1,993 (6.3%) | 1,980 (6.2%) |
| Missing | 501 | 401 | 483 | 87 | 80 | 86 |
| **Cardiovascular diseases** |  |  |  |  |  |  |
| No | 14,038 (80.7%) | 14,852 (85.3%) | 15,176 (87.2%) | 29,477 (93.6%) | 30,654 (96.3%) | 31,070 (97.5%) |
| Yes | 3,363 (19.3%) | 2,550 (14.7%) | 2,226 (12.8%) | 2,011 (6.4%) | 1,181 (3.7%) | 800 (2.5%) |
| Missing | 6 | 5 | 5 | 0 | 0 | 0 |
| **Diabetes** |  |  |  |  |  |  |
| No | 15,772 (90.8%) | 16,423 (94.5%) | 16,567 (95.3%) | 30,879 (98.1%) | 31,575 (99.2%) | 31,714 (99.5%) |
| Yes | 1,600 (9.2%) | 954 (5.5%) | 811 (4.7%) | 609 (1.9%) | 260 (0.8%) | 156 (0.5%) |
| Missing | 35 | 30 | 29 | 0 | 0 | 0 |
| **Cancer** |  |  |  |  |  |  |
| No | 15,748 (90.8%) | 16,006 (92.3%) | 16,254 (93.7%) | 29,192 (92.7%) | 29,761 (93.5%) | 29,844 (93.6%) |
| Yes | 1,596 (9.2%) | 1,343 (7.7%) | 1,099 (6.3%) | 2,296 (7.3%) | 2,074 (6.5%) | 2,026 (6.4%) |
| Missing | 63 | 58 | 54 | 0 | 0 | 0 |

# Supplementary Figure 1. Flowchart of participant inclusion

NAKO population with accelerometry

N = 72,553

UK Biobank population with accelerometry

N = 103,578

NAKO population for mortality assessment

N = 52,352

UK Biobank population for mortality assessment

N = 95,193

No follow-up data

N = 4 (NAKO)

Lost-to-follow-up before accelerometry (UK Biobank)

N = 4

Age <40 or >74 years

N = 12,766 (NAKO)

N = 1,397 (UK Biobank)

NAKO population with valid data

N = 65,122

UK Biobank population with valid data

N = 96,594

Poor accelerometry data

N = 7,431 (NAKO)

N = 6,984 (UK Biobank)

# Supplementary Figure 2. Directed acyclic graph for confounder selection

# Supplementary Figure 3. Correlation matrix for all physical activity exposures

AvgAcc: average acceleration (overall physical activity volume; milli-*g*); LPA: light physical activity (minutes per week); MPA: moderate physical activity (minutes per week); VPA: vigorous physical activity (minutes per week)

# Supplementary Figure 4. Exclusion of 1 to 5 years of follow-up

# Supplementary Figure 5. Main results without adjustment for body mass index, grip strength, cardiovascular disease, diabetes, and cancer

# Supplementary Figure 6. Main results after exclusion of prevalent cardiovascular disease, diabetes, and cancer

# Supplementary Figure 7. Main results after restriction to participants who never smoked

# Supplementary Figure 8. Main results with additional adjustment for season of accelerometer wear

# Supplementary Figure 9. Main results under mutual adjustment for light, moderate, and vigorous physical activity
